# 

**Published:** 1963-10

**Authors:** 


					4)
h2S
October 19G3
Journ
INCORPORATING THE MEDICAL JOURNAL OF THE SOUTH WEST
Vol 78 (iv) No 290

				

